# DNA Methylation Regulator-Meditated Modification Patterns Define the Distinct Tumor Microenvironment in Lung Adenocarcinoma

**DOI:** 10.3389/fonc.2021.734873

**Published:** 2021-09-06

**Authors:** Didi Yuan, Zehong Wei, Yicheng Wang, Fang Cheng, Yujie Zeng, Li Yang, Shangyu Zhang, Jianbo Li, Renkuan Tang

**Affiliations:** Department of Forensic Medicine, College of Basic Medicine, Chongqing Medical University, Chongqing, China

**Keywords:** lung adenocarcinoma (LUAD), DNA methylation, tumor microenvironment, immunotherapy, mutation burden

## Abstract

**Background:**

Epigenetic changes of lung adenocarcinoma (LUAD) have been reported to be a relevant factor in tumorigenesis and cancer progression. However, the molecular mechanisms responsible for DNA methylation patterns in the tumor immune-infiltrating microenvironment and in cancer immunotherapy remain unclear.

**Methods:**

We conducted a global analysis of the DNA methylation modification pattern (DMP) and immune cell-infiltrating characteristics of LUAD patients based on 21 DNA methylation regulators. A DNA methylation score (DMS) system was constructed to quantify the DMP model in each patient and estimate their potential benefit from immunotherapy.

**Results:**

Two DNA methylation modification patterns able to distinctly characterize the immune microenvironment characterization were identified among 513 LUAD samples. A lower DMS, characterized by increased CTLA-4/PD-1/L1 gene expression, greater methylation modifications, and tumor mutation burden, characterized a noninflamed phenotype with worse survival. A higher DMS, characterized by decreased methylation modification, a greater stromal-relevant response, and immune hyperactivation, characterized an inflamed phenotype with better prognosis. Moreover, a lower DMS indicated an increased mutation load and exhibited a poor immunotherapeutic response in the anti-CTLA-4/PD-1/PD-L1 cohort.

**Conclusion:**

Evaluating the DNA methylation modification pattern of LUAD patients could enhance our understanding of the features of tumor microenvironment characterization and may promote more favorable immunotherapy strategies.

## Introduction

The global DNA methylation is strongly associated with growth selection and uncontrolled cell proliferation in multiple cancer types (1, 2). Among the epigenetic mechanisms of the mammalian genome, DNA methylation is catalyzed by series of DNA methyltransferases catalyzing the transfer of a methyl group from *S*-adenyl methionine to the C5 position of a cytosine residue to form 5-methylcytosine at CpG sites (3). The expression and function of those methyltransferases has been reported to be partly responsible for immunomodulation and might have an impact on DNA methylation modification patterns in human cancers (4, 5).

Lung adenocarcinoma (LUAD) is the most common form of lung cancer and is responsible for the cancer deaths worldwide (6). However, the overall survival time of LUAD patients remains short despite the enormous research efforts for the development of effective diagnostic techniques and therapeutics. Recently, as a result of an increasing number of studies focusing on tumor immune cell infiltrations and DNA epigenetic modification, crucial immune cell types and methyltransferase subsets in tumor growth, metastasis, and outcome have gradually been identified (7–10). The integrated analysis of the composition of the immune cells in both LUAD tumors and paired normal adjacent tissues has revealed that a deeper exploration of immune signatures or biomarkers in the tumor microenvironment (TME) could play an essential role in revealing the potential oncogenic mechanism in LUAD (11). Moreover, the immunotherapeutic efficacy of several immune checkpoint inhibitors (ICIs) (anti-CTLA4/PD-L1/PD-1, 8) has widely assessed and has achieved a notable response in tumor treatment, including LUAD (12–14). Further studies have also proposed that the identification of altered epigenetic methylation patterns may represent a valuable diagnostic approach toward novel therapeutic strategies for preventing and treating LUAD. For instance, Yang et al. reported that the downregulation of methyltransferases DNMT3A and MBD4 could promote ALDH2 expression, reducing the probability of bone metastasis in LUAD patients (15). Forloni et al. found that the inhibition of the demethylase TET oncogene family member 1 (TET1) could induce epigenetic silencing of antitumor genes in the oncogenesis EGFR signaling pathway, indicating that dysregulated DNA methylation probably played a major role in tumorigenesis (16). However, the global profiles of DNA methylation regulators based on the correlation between immune microenvironment and immunotherapy of LUAD samples have not been fully evaluated (12, 17).

In this study, we collected and integrated the clinical information and genomic data of 513 patients from The Cancer Genome Atlas (TCGA) LUAD cohort and comprehensively evaluated the TME characteristics represented by distinct patterns of DNA methylation modifications. We finally identified two independent DNA methylation patterns (DMPs) by unsupervised consensus clustering the expression of 21 DNA methylation regulator-related genes, which we defined as methylation regulators. The immune cell-infiltrating properties of two DMPs were highly consistent with immune-noninflamed or immune-inflamed phenotype, respectively. We then constructed a DNA methylation score (DMS) system to investigate the efficacy of DNA methylation modification patterns in individual patients and estimated their immunotherapeutic value in several clinical trials.

## Materials and Methods

### Data Collection and Preparation

The workflow of this study is presented in **Figure S1A**. Gene expression data and complete clinical annotations of LUAD samples were retrospectively collected from publicly available datasets of the Gene-Expression Omnibus (GEO) and TCGA databases. A total of six GEO lung adenocarcinoma cohort somatic mutation, copy number variation (CNV), and clinical data, including tumor stage, age, sex, and overall survival times/states were obtained from TCGA databases (GSE116959, GSE58772, GSE99995, GSE68571, GSE68465, and GSE26939) and one TCGA-LUAD cohort were enrolled for further analysis. For the GEO microarray data, the normalized matrix files were directly downloaded. As for TCGA cohort, the RNA sequencing data (FPKM value) for gene expression was downloaded from the Genomic Data Commons and then transformed into transcripts per kilobase million (TPM) format. Batch effects among GEO datasets were corrected using the “ComBat” algorithm of the sva package. The baseline information of all eligible datasets is summarized in **Table S1**.

### Consensus Molecular Clustering of 21 DNA Methylation Regulators

We collected DNA methylation regulator-related studies, and a total of 21 regulator genes (including “writers’: DNMT1, DNMT3A, DNMT3B; “easers”: TET1, TET2, TET3; and “readers”: MBD1, MBD2, MBD3, MBD4, TDG, SMUG1, UHRF1, UHRF2, ZBTB4, ZBTB24, ZBTB33, ZBTB38, NSUN2, MGMT, DMAP1) were extracted for DNA methylation modification pattern identification **(** **Table S2**). The ConsensusClusterPlus algorithm was employed to conduct unsupervised clustering of individual tumor samples with the gene expression profiles of 21 DNA methylation regulators (18). The cluster assignments were stable when *k* = 2.

### Gene-Set Variation Analysis and Functional Annotation

We performed Gene-Set Variation Analysis (GSVA) enrichment analysis with “GSVA” R packages to study the differed biological pathway between different DNA methylation modification patterns in cancer samples (19). The Hallmarker gene-set of “c2.cp.kegg.v7.4.symbols” was retrieved from the MSigDB database (20). Adjusted *p*-values <0.05 were considered statistically significant for GSVA analysis. Gene ontology (GO) and pathway annotation for DNA methylation pattern-related genes were performed using the R package “clusterProfiler” with a cutoff value of *p* < 0.05.

### Immune Cell Infiltration Estimation

The Single Sample Gene-Set Enrichment Analysis (ssGSEA) was implemented to determine variations in immune leukocyte subtype abundance between different DMP clusters using the R package “GSEAbase.” Subsequently, the abundances of 22 immune cell types for each tumor specimen were further identified by estimating relative subsets of RNA transcripts (CIBERSORT; https://cibersort.stanford.edu/) using the gene expression profile of LUAD cancer (21).

### Identification of Differentially Expressed Genes and DMS Construction

We used three R packages (“limma,” “edgeR,” and “Deseq2”) to identify the DNA methylation modification-related differentially expressed genes (DEGs) across distinct DMP phenotypes. Univariate Cox model analysis was performed to calculate their association with overall survival and to extract prognostic DEGs to construct a scoring system. We then conducted principal component analysis (PCA) using the identified DEG prognostic genes, and PCA 1–2 components were selected to act as signature scores to construct DNA methylation-relevant gene signature, which we defined as the DMS. This method mainly focused on evaluating the score for each patient in the dataset with the largest group of well-correlated (or anticorrelated) genes. We constructed the DMS using an algorithm similar to the GGI: DMS = Σ(PC1*_i_* + PC2*_i_*), where *i* stood for the relevant gene expression of the selected set (22, 23).

### Immunotherapy Dataset Collection

We searched the immunotherapeutic characteristics of the TCGA-LUAD patients using the Cancer Immunome Database (TCIA), which is a publicly available dataset containing corresponding clinical pathology information (24). The immunotherapeutic GEO datasets were included in this study: GSE135222 (anti-PD-1/PD-L1 treatment) (25) and GSE91061 (anti-CTLA-4/PD-1 treatment) (26). We also retrieved IMvigor210 datasets containing data on atezolizumab treatment and extracted the relative gene expression profiles and clinical notes using the R “IMvigor210CoreBiologies” package (27). The raw count data were then transformed into TPM value format.

## Results

### Landscape of DNA Methylation Regulators in LUAD

**Figure 1:**
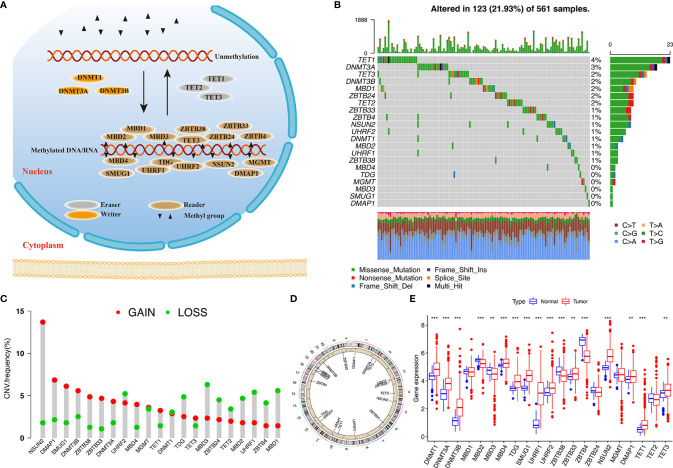
Landscape of genetic variation of DNA methylation regulators in LUAD. **(A)** Summary of 21 DNA methylation regulators and their potential biological processes. **(B)** Mutation landscape of 21 DNA methylation regulators in 561 LUAD patients. Individual patients are presented in the upper column. Mutation frequency of each regulator are represented in the right bar plot. **(C)** The CNV frequency of 21 DNA methylation regulators in TCGA cohort. **(D)** The location of 21 regulators on chromosomes. **(E)** Differences in gene expression levels of 21 regulators between normal and tumor patients in TCGA cohort. **p < 0.01, ***p < 0.001.

In this study, 21 DNA methylation regulators were collected from the published data. **Figure 1A** displays the dynamic reversible process of RNA methylation mediated by regulators and their corresponding potential biological functions. We further analyzed the genetic alterations of DNA methylation regulators in LUAD. Among the 561 cases, 123 samples (21.93%) harbored genetic alterations of DNA methylation regulators, primarily including nonsense or missense mutations. TET1 (4%) had the highest mutation frequency, followed by DNMT3A, TET3, and DNMT3B (**Figure 1B**). A mutation cooccurrence pattern across all DNA methylation regulators was examined, and significant gene mutation relationships were identified between DNMT1 and ZBTB4, MBD4 and NSUN2, and DNMT1 and UHRF1 (**Figure S1B**). Further analysis of CNV alteration frequency in the 21 regulators showed a prevalence of CNV mutations. Among these, NSUN2, DMAP1, SMUG1, DNMT3B, ZBTB33/38, and DNMT3A showed relatively higher amplification frequency in terms of CNV, while MBD1/2/3, ZBTB4/24, UHRF1, TDG, and TET2 had a widespread frequency of copy number deletion variants (**Figure 1C**). The location of CNV among the DNA methylation regulators on chromosomes is shown in **Figure 1D**. To investigate whether these genetic variants influenced the gene expression of regulators in cancer samples, we compared the expression of the 21 DNA methylation regulators between normal and LUAD samples and found that regulators with significant genetic mutation as well as CNV amplification were significantly higher expressed in cancer groups (**Figures 1B–E**), indicating that the regulator gene mutation and the alternation of CNV could be risk factors leading to the multiperturbation of the translation of DNA methylation regulators. Univariable and multivariable Cox proportional hazards regression analyses were performed to evaluate the association between regulators and patient survival. The Forest plot and Kaplan-Meier curve showed that DMAP1 and MGMT were upregulated in tumor specimens and could be considered protective factors associated with prolonged survival time, while ZBTB38 and MBD2/3 downregulation in the tumor group was recognized as a risk factor with worse overall survival (**Figures S1C–H**), indicating that the unbalance expression of DNA methylation regulators could contribute to tumorigenesis. These analyses demonstrated that the crosstalk between DNA methylation regulators in the genomic and transcriptomic landscape has a potential role in LUAD occurrence and complexity.

### DNA Methylation Modification Patterns in TCGA Cohort

**Figure 2:**
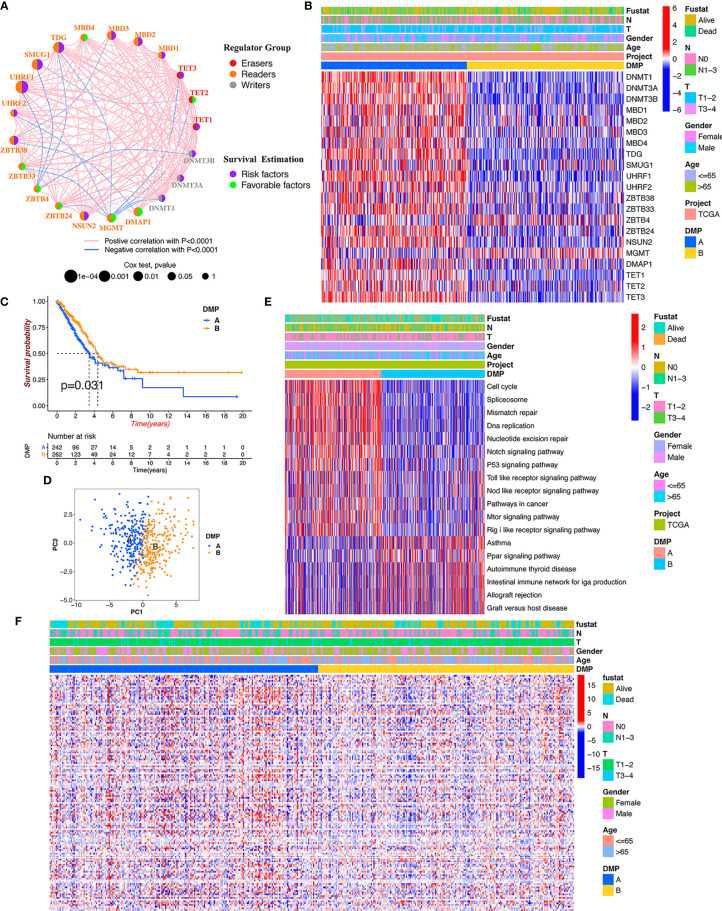
DNA methylation modification pattern and relevant biological characterization. **(A)** The interaction and prognosis of 21 DNA methylation regulators in LUAD. The size of the node represents the prognostic value of each regulator; the left semicircle represents three distinct regulator types; the right semicircle represents the prognostic factors for LUAD. The line represents the correlation among regulators: red line, positive correlation; blue line, negative correlation. **(B)** The consensus clustering of 21 regulators in 513 TCGA-LUAD samples. Red represents higher expressed regulators. Blue represents lower expressed regulators. **(C)** Survival analysis of distinct DNA methylation patterns in TCGA cohort. **(D)** Principal component analysis for the gene expression of DNA methylation regulators to discriminate DNA methylation modification patterns. **(E)** GSVA enrichment analysis identifying biological pathways of each DNA methylation pattern. DMP, DNA methylation pattern. **(F)** The heatmap analysis of DNA methylation level evaluation in different DNA methylation patterns.

To determine the pattern of DNA methylation modification mediated by 21 regulators, the TCGA-LUAD cohort and their corresponding clinical data were prepared for analysis (**Table S3**). A comprehensive landscape of the interactions and the prognostic significance of the 21 regulators was visualized using the conetwork plot (**Figure 2A**). We dissected the relationship among those regulators and found that most regulators showed a positive correlation with each other, which is consistent with the above analysis. We also demonstrated that the expression of UHRF1, SMUG1, TDG, and MBD1/2/3 presented the most significant positive correlation with other regulators and might be the risk factors for carcinoma formation. Notably, the readers, such as MGMT and ZBTB4, showed a remarkably negative relationship with the other regulators. Thus, these findings indicated that crosstalk among DNA methylation regulators may play a critical role in the onset of LUAD.

We continued to conduct unsupervised clustering based on the expression of 21 DNA methylation regulators to identify DNA methylation modification patterns in LUAD samples, and two distinct clusters were accordingly obtained *via* the ConsensusClusterPlus package in R software, including 246 cases in cluster A and 267 cases in cluster B (**Figure 2B**). We renamed those clusters as DNA methylation pattern (DMP-A and DMP-B) (**Table S4**). Survival analysis for the two DNA methylation modification clusters showed that DMP-B had a relatively better prognosis in the TCGA cohort (**Figure 2C**). In addition, we conducted heatmap analysis of the relative expression of 21 DNA methylation regulators between distinct DNA methylation modification patterns and observed that most regulators were markedly elevated in the DMP-A cluster (**Figure 2B**). Principal component analysis (PCA) also indicated that two DMP clusters were completely segregated, indicating that they could be easily distinguished *via* the gene expression pattern of the 21 regulators (**Figure 2D**). The heatmap analysis of the tumor methylation status determined that the DMP-B cluster presented a low-methylation epigenotype with worser clinical outcomes (**Figure 2F**). These results demonstrated that altered expression of DNA methylation regulators could affect patient survival by regulating tumor methylation levels, which contributes to the high heterogeneity of LUAD.

### TME Cell Infiltration Characteristics of the Two Distinct DMPs in the TCGA Cohort

**Figure 3:**
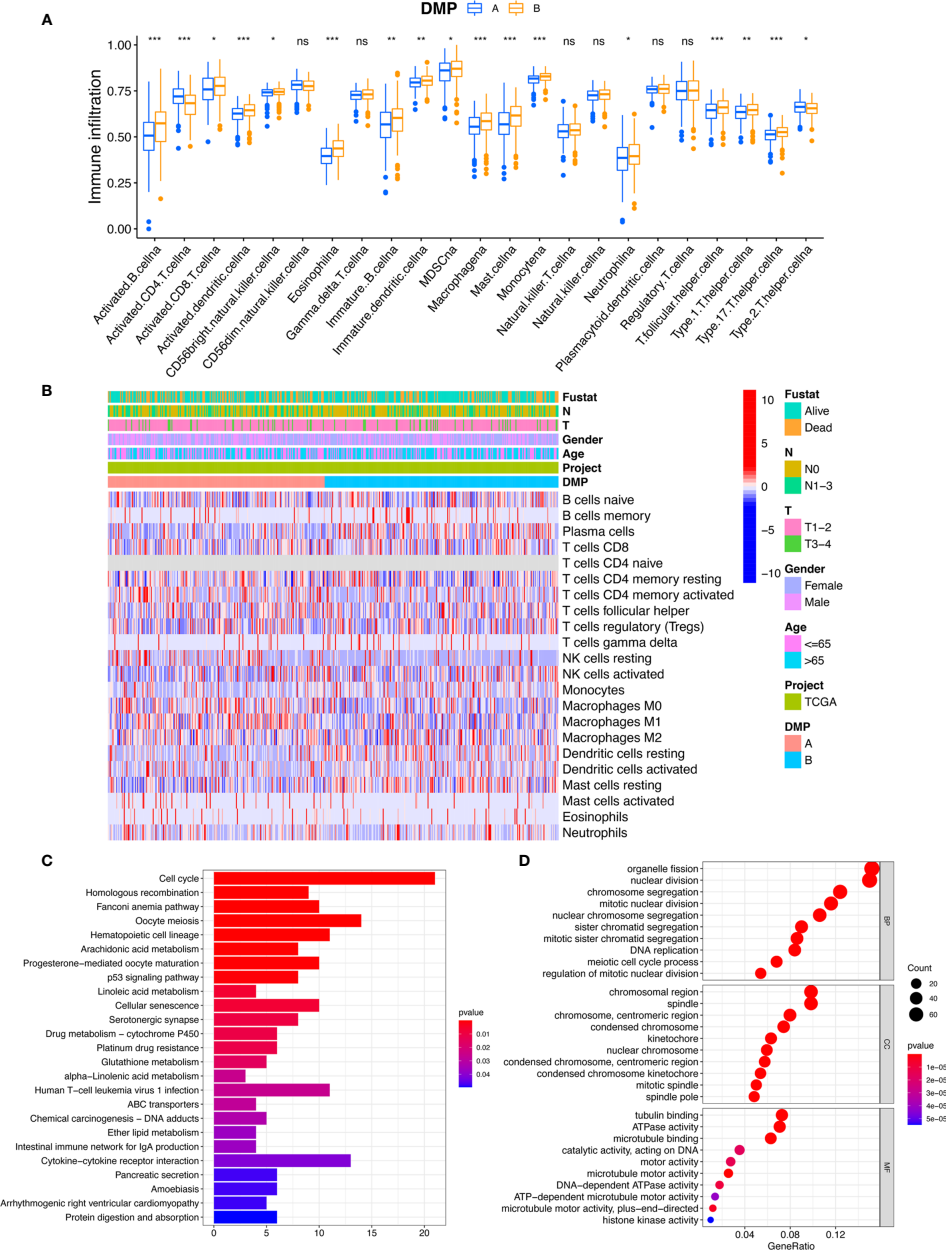
TME characteristics and transcriptome traits of DNA methylation modification patterns. **(A)** The abundance of immune infiltration cells in two DMP clusters using ssGSVA algorithm. *p < 0.05, **p < 0.01, ***p < 0.001. **(B)** The CIBERSORT heatmap of tumor immune-infiltrating cells in TCGA cohort. Clinical data regarding survival state, tumor stage, gender, age, and DMP cluster were annotated above. Red represents the higher fraction of immune cells in each sample; blue, the lower fraction. **(C, D)** Functional annotation for DMP-related genes using GO and KEGG enrichment analyses. The color depth of the KEGG barplots represents the enriched gene count. ns, no significance.

To further explore differences in biological behaviors among the two DNA methylation modification patterns, we performed GSVA enrichment analysis. As shown in **Figure 2E** and **Table S5**, DMP-A was mainly presented a CD4+ immune regulator and stromal activation phenotype, associated with many related pathways, such as the cell cycle, DNA replication, Toll-like receptor, and Nod-like receptor signaling pathways in cancer. Whereas, DMP-B represented enriched pathways associated with immune/inflammation hyperactivation including the activation of the PPAR signaling pathway and the autoimmune response, including asthma, autoimmune thyroid disease, allograft rejection, and graft-*versus*-host disease. Furthermore, we conducted a TME cell infiltration analysis using the ssGSVA algorithm and demonstrated that DMP-A showed a relatively lower proportion of immune cell infiltration and exhibited a shorter survival time (**Figures 2B**, **3A**). However, DMP-B did present a matching survival advantage and significantly elevated innate immune cells including activated B cells, MDSCs, macrophages, mast cells, natural killer cells, and activated CD8+ T cells and so on. The CIBERSORT analysis also indicated that DMP-B was markedly enriched in immunoactive cells, such as M2 macrophages, memory B cells, plasma cells, and Tregs, which is coherently indicative of a better prognosis (**Figures 2B**, **3B**). Previous studies have reported that the immune-excluded phenotype in tumors also presented immune cells limited to the stroma surrounding the tumor cell nests (28). Therefore, we suspected that the stromal activation and low immune cell density in DMP-A pattern caused the suppression of antitumor response, which was also validated in the GEO LUAD cohort (GSE116959, GSE58772, and GSE99995) (**Figure S2I**). Altogether, these results indicated that LUAD patients could be classified into two immune phenotype groups, among which the DMP-A pattern represented a noninflamed phenotype (immune-excluded or immune-deserted trait) characterized by stromal activation and weakened immune regulator status, whereas the DMP-B pattern represented an immune-inflamed phenotype characterized by immune/inflammation hyperactivation and multicomponent immune cell infiltration in the TME.

### DMP Phenotype-Related DEGs and Functional Annotation

**Figure 4:**
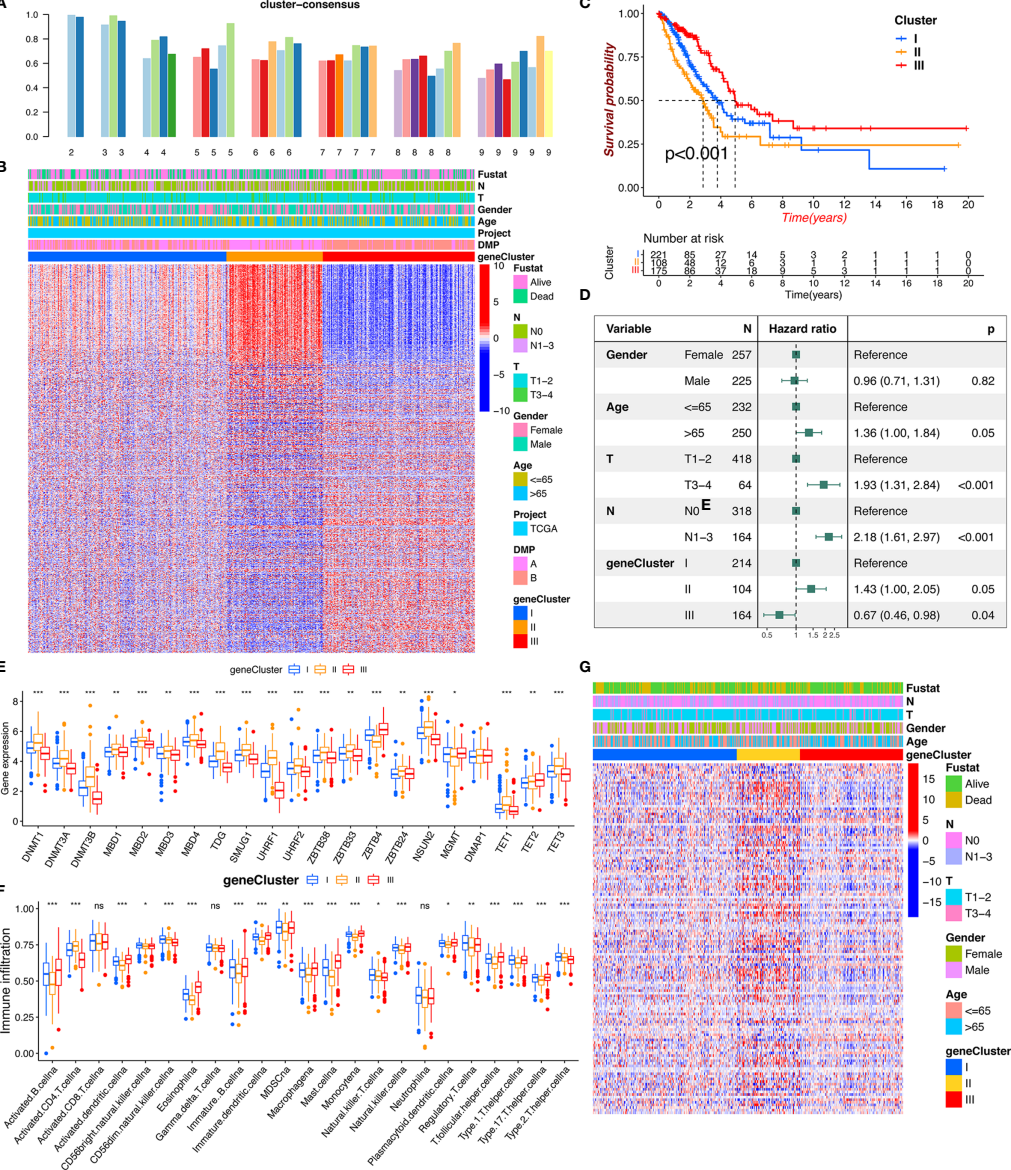
DMP phenotype-related DEGs and the clinical characterization of gene clusters. **(A)** Cluster-consensus (CLC) plot of unsupervised clustering showed the consensus scores of subcluster at each (*k*) **(B)** Unsupervised clustering of 586 DNA methylation-related genes in the TCGA LUAD cohort classifying patients into three distinct subclusters, renamed as gene cluster I/II/III, respectively. The clinical annotation information including survival state, tumor stage, sex, age, and DMP cluster, as well as gene cluster were enrolled. **(C)** Kaplan-Meier plotter estimated the survival outcome of the different gene clusters. **(D)** Multiple Cox regression analysis estimated the clinical prognostic value between gene clusters in the TCGA cohort. The length of the horizontal line represents the 95% confidence interval for each group. **(E)** The expression level of 21 regulators in distinct gene clusters. **(F)** The immune cell infiltration fraction in three gene clusters using the ssGSEA algorithm. The asterisks represented as the statistically significant *p*-values: *p < 0.05, **p < 0.01, ***p < 0.001. **(G)** The heatmap analysis of DNA methylation status evaluation for each gene cluster. ns, no significance.

To further explore the characteristics of the DMP in the LUAD cohort, a total of 2,720 DNA methylation regulator pattern related to the DEGs were obtained and subjected to conduct enrichment analysis. The KEGG pathway related to the cell cycle and the p53 signaling pathway were also significantly enriched in DMP-related gene set (**Figure 3C**). The results of GO terms showed that those genes were mainly enriched in the cell cycle process and DNA replication (**Figure 3D**). We subsequently selected 792 prognostic genes (*p* < 0.05) *via* univariate Cox model analysis (**Table S6**). Based on the expression of the 792 representative genes, unsupervised clustering analysis was performed to classify LUAD patients into three distinct clusters, namely, DNA methylation gene cluster I/II/III (**Figures 4A, B**; **Figures S2A–E**). The heatmap and survival analysis showed that patients in gene cluster III were characterized by earlier clinical stage and better prognosis, while patients in gene cluster II were characterized by advanced staging and worser survival outcome (**Figures 4B, C**). In addition, multivariate Cox regression analysis indicated that the overall survival between the gene cluster gene signatures remained statistically significant after considering multiple factors, including age, tumor stage (T and N), and sex (**Figure 4D**), indicating that the identified gene clusters could represent independent factors of prognosis. We also noted that the gene expression of the 21 regulators exhibited significant differences among the three gene clusters, and in particularly patients in gene cluster III were related to the higher expression of protective factors (TET2/MGMT), which is in line with the findings regarding DNA methylation modification patterns (**Figure 4E**). The patients in gene cluster III also presented higher immune cell infiltration, indicating a phototype of immune activation. While the levels of almost all immune cells in gene cluster II were relatively lower than in other groups, these presented a phenotype of immune suppression (**Figure 4F**). We further evaluated the DNA methylation status of the gene clusters, and results indicated that the gene cluster II exhibited a hypermethylation status, whereas the gene cluster III presented a weaker methylation status (**Figure 4G**). All these results also confirmed that the perturbed DNA methylation regulator genetic expression was highly correlated with different immune responses, genetic methylation levels, and clinical outcomes and contributed to LUAD heterogeneity, which was consistent with the previous findings.

### Construction of DMS and Exploration of Clinical Relevance

To accurately evaluate the DNA methylation status of individual patients with LUAD, we developed a scoring scheme termed the DMS based on the expression of 792 DNA methylation-related genes.

**Figure 5:**
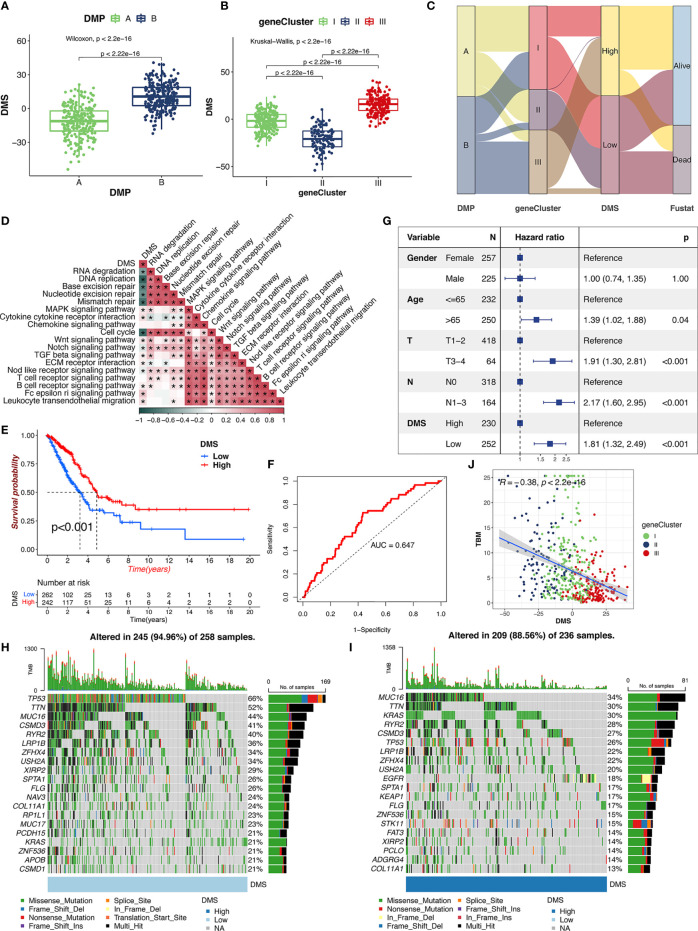
The clinical characteristics of DMS. **(A)** Distribution of DMS in distinct DMPs in TCGA cohort. The Wilcoxon text, *p* < 0.001. **(B)** Distribution of DMS in distinct gene clusters in TCGA cohort. The Kruskal-Wallis text, *p* < 0.001. **(C)** The alluvial diagram of the relationship among DMPs, gene clusters, DMS, and prognosis. **(D)** The correlations between DMS and the enriched gene signal pathways in TCGA cohort using the Spearman’s test. **(E)** Survival analysis of high- and low-DMS groups in TCGA cohort. Log-rank text, *p* < 0.001. **(F)** The predictive value of the DMS signatures in TCGA cohort. **(G)** Multivariate Cox regression analysis for DMS in TCGA cohort. **(H, I)** The landscape of tumor somatic mutation in TCGA cohort evaluated between low **(H)** and high **(I)** DMS. **(J)** Correlations between DMS, TBM, and gene cluster in TCGA cohort.

The DMP-B pattern had a higher level of DMS compared with patients in DMP-A (**Figure 5A**). Notably, gene cluster III showed the highest level of DMS, followed by gene clusters I and II (**Figure 5B**). With an optimum cutoff value of 2.17 determined by the survminer package, we divided LUAD patients into high- and low-DMS groups. The alluvial diagram summarized the attribute changes of patients according to the DNA methylation regulator pattern, gene cluster, and DMS groups (**Figure 5C**). We then examined the correlation between biological processes, immune cell infiltration, and the level of DMS signatures using Spearman’s analysis. The DMS signatures were significantly negatively correlated with cell cycle and DNA/RNA repair signatures but were positively correlated with immune activation and stromal-relevant pathways (**Figure 5D**; **Figure S2F**). In addition, we calculated the distribution differences of tumor genome somatic mutations between the DMS groups using the R “maftools” package. As we expected, the low-DMS group with shorter survival time presented significantly higher tumor mutational profiles than the high-DMS group, consistent with recent findings that gene alterations correlated highly with tumor invasion and cell proliferation. Furthermore, survival analysis revealed that the DMS-low patients were significantly correlated with a worse prognosis in the TCGA cohort (*p* < 0.001, **Figure 5E**), which was further validated by the ROC curves (AUC = 0.652, **Figure 5F**). The multivariate Cox regression model confirmed the DMS factor could stand as an independent prognostic biomarker for evaluating patient outcomes in the TCGA-LUAD cohort (HR, 2.03 [95% confidence interval, 1.34–3.08], *p* < 0.001, **Figure 5G**). To further verify this DMS model, we also performed multivariate Cox regression model and prognosis analysis using meta-GEO cohorts (GSE68465, GSE68571, and GSE26939), which provided further support that DMS was a significant prognostic factor for predicting patient outcomes (**Figures S2G, H**). The analysis of tumor mutation burden (TMB) confirmed that the low-DMS cluster was significantly correlated with a higher TMB (**Figures 5H, I**). The mutational landscape of significantly mutated genes showed that most genes (including TP53 and TTN) had higher somatic alteration rates in the lower-DMS group, whereas KRAS had a higher somatic alteration rate in the higher-DMS group. We further observed that there was a markedly negative correlation between the TMB score and DMS (*R* = −0.48, *p* < 0.001), demonstrating the crosstalk between the DMS and genetic mutation evaluation (**Figure 5J**). Meanwhile, the methylation heatmap analysis revealed that the higher-DMS group exhibited hypomethylation compared with the lower-DMS group (**Figure S2J**). These results demonstrated that the DMS could represent the DNA methylation modification patterns and comprehensively could reflect genomic variation modifications, as well as effectively predict the prognosis of LUAD patients.

### The Role of DMS in Predicting Immunotherapy

**Figure 6:**
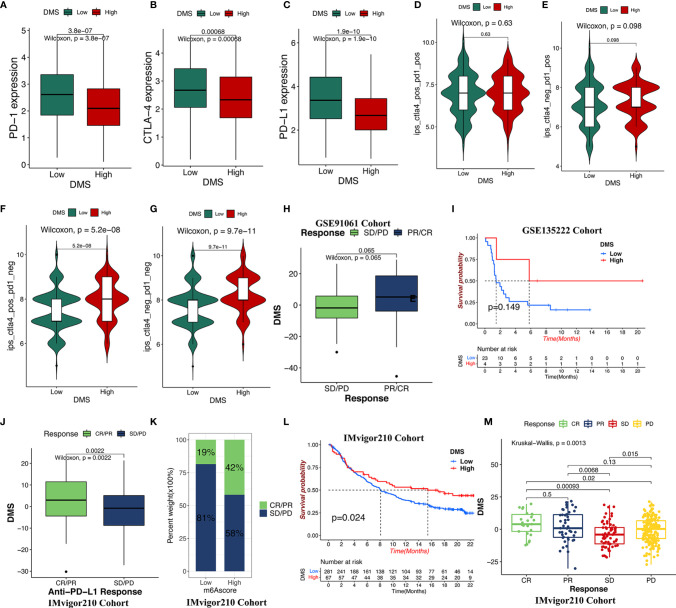
The role of DMS in anti-CTLA-4/PD-1/PD-L1 immune treatment cohort. **(A–C)** The relative expression of immune checkpoints between high DMS and low DMS in TCGA cohort. **(D–G)** Immunotherapeutic benefits of anti-CTLA-4/PD-1 treatment in TCGA cohort. **(H)** Difference in the DMS for anti-CTLA-4/PD-1 clinical responses in the GSE91061 dataset. CR/PR, complete response/partial response; SD/PD, stable disease/progressive disease. **(I)** Progression-free survival analysis for distinct DMS groups in GSE135222 cohort. **(J, K)** Difference in the DMS for anti-PD-L1 clinical response in the IMvigor210 cohort. **(L)** Survival analysis for low- and high-DMS groups in the IMvigor210 cohort. **(M)** The clinical response of patients treated with anti-PD-L1 according to DMS groups.

Significant progress has been made to identify the effective immune-related signatures that correlated with response to antitumor therapy, particularly represented by TMB and ICIs. Our analysis found that the gene expression of the CTLA-4/PD-1/PD-L1 in TCGA-LUAD cohort was significantly increased in the DMS-low group (**Figures 6A–C**). Considering the high correlation across DMS levels with the immune response, we explored whether the DMS system could predict the patients’ response to ICI treatment in two independent immunotherapy cohorts treated with CTLA-4/PD-1/PD-L1 antibody inhibitors. In the TCIA-LUAD cohort (anti-CTLA-4/PD-1 treatment), patients classified as the DMS-high group exhibited a significantly clinical response (**Figures 6D–G**), indicating the immunotherapeutic benefits of CTLA-4/PD-1 antibody treatment in DMS-high patients. In the anti-CTLA-4/PD-1/PD-L1 GEO cohort (GSE91061 and GSE135222), higher DMS in tumor patients was associated with a stronger immune response and higher therapeutic benefits (**Figures 6H, I**). The progression-free survival analysis showed that higher-DMS patients exhibited prolonged survival (**Figure 6I**). A similar result was also identified in the IMvigor210 cohort (anti-PD-L1 treatment) (**Figures 6J–M**). Those results demonstrated that the DMS signature system was strongly associated with the tumor immune response and might contribute to predicting the efficacy value of the anti-CTLA-4/PD-1/PD-L1 immunotherapy.

## Discussion

Increasing evidence has indicated that aberrant DNA methylation might increase genome instability by silencing of notable antioncogenes by methylated modifications, resulting in TME alterations, CNV, and biological process conversion (29, 30). However, the global modulation of DNA methylation modification in the immune contexture of LUAD patients remains to be comprehensively recognized. Thus, the identification of distinct DNA methylation modification patterns in the tumor immune microenvironment could provide a useful evaluation of the correlation between DNA methylation on the immune-related response and may assist in achieving more effective immunotherapy resolution.

Herein, following unsupervised clustering analysis of the gene expression of 21 regulators, we defined two different DMPs characterized by distinct immune phenotypes in the tumor immune microenvironment. DMP-A–clustered patients presented with hypermethylation had a lower proportion of immune cells, characterized by the suppression of the immune cell-infiltrating response in tumor cells and a noninflamed (immune-excluded/deserted) phenotype, which corresponded to a worser survival prognosis. In contrast, the DMP-B–clustered individuals presented with hypomethylation was characterized by the activation of abundant immune effector/pathways and the presence of multiple immune cell infiltrations in the TME, which exhibited an immune-inflamed phenomenon, corresponding to significant survival benefits. Analysis of the tumor immunophenotype in patients with solid tumors has also widely supported the view that the immune environment plays a central role in the pathogenesis of tumorigenesis and metastasis and harbored important clinical implications for effective immunotherapy outcomes (27, 31–33). The available literature to date has reported that the immune-excluded phenomenon is largely reflected by hyper-invigorating stromal cells surrounding tumor cell nests, categorized as an immune-inflamed phenotype (34, 35). Stroma with lower expression of immune markers (tumor-infiltrating lymphocytes and CD8+ T cells) could suppress the antitumor signatures and interfere with the penetration of immune cell infiltration into the tumor parenchyma, consistent with the poorer survival in the DMP-A immune phenotype (36–38). The immune-inflamed phenotype was represented by a higher density of immune T cells, enrichment of abundant immune signal pathways, and the presence of preexisting immune infiltration in an antitumor microenvironment (34, 39). Recapitulating our analysis of the TME immune-infiltrating characteristics using the ssGSEA and CIBERSORT algorithm with our proposed DMP clusters confirmed the above definition of immune classification. In addition, previous reports have also shown that DNA hypomethylation is associated with immune signaling activation (40), which further supported our findings in this study.

To further elucidate the characteristics of transcriptome traits in DMP, the DEGs of distinct DNA methylation modification patterns were obtained and selected as the DNA methylation signature genes, which were concurrently found to be significantly associated with the cell cycle and DNA replication biological behaviors. Based on the mRNA expression levels of these DEGs, three DNA methylation gene clusters with different survival prognosis and immune-infiltrating features were identified, indicating there were indeed different clinical immune phenotypes associated with LUAD (11, 41). In addition, we established a methylation-based scoring system to quantify the DNA methylation modifications of individual patients, which helped to define the DMP signature, thus yielding greater scientific insight into the complex mechanisms of tumorigenesis and progression (27, 31, 32). As expected, the DMP-A group was associated with gene cluster I/II and was characterized by the presence of immune suppression and a higher methylation phenotype and a lower DMS, which corresponded to a shorter survival time. The multivariate analysis of the LUAD cohort confirmed the DMS system could be an independent factor for patient prognosis. Altogether, the above results indicated that there was indeed a higher heterogeneity of DNA methylation modifications in the tumor immune/alteration microenvironment of LUAD (42).

Consistent with the previous analysis, the DMS, which was negatively correlated with TMB, was found to be markedly enriched in the immune activation and stromal-relevant pathways, underlining the pivotal role of stromal immune activation in resistance to immune checkpoint therapeutics (43, 44). Our study revealed that cancer drug resistance was not only accompanied by the hyperactivation of various stromal pathway associated with TGF beta, MAPK, and Wnt signaling pathway but also correlated to gene repair and cell cycle processes. For instance, the inhibition of the TGF beta pathway was reported to induce durable responses to PD-1/PD-L1 blockade in tumor models (45, 46). Previous studies also demonstrated that the cancer epithelial-mesenchymal transition is frequently activated by TGF-β, Wnt, Notch, and MAPK signaling pathways, which are the major factors promoting metastasis and notorious invasion of cancer cells (47, 48). Furthermore, recent studies searching for the effective immunotherapies in cancer and found that ICIs were successful cancer treatments, particularly in metastatic urothelial cancer and melanoma where anti-CTLA-4/PD-1/PD-L1 antibodies have found widespread application (27, 49, 50). However, using our data, we evaluated the therapeutic value of the DMS in the immune checkpoint (CTLA-4/PD-1/PD-L1) treatment cohorts and showed the opposite clinical benefit to immune inhibitors. Compared with the lower-DMS group, the higher-DMS group, which presented a smaller proportion of genetic mutations and methylation, exhibited a stronger antitumor immune response and benefit of ICI treatment. Meanwhile, TP53 and TTN mutations in the lower-DMS subgroup showed a larger proportion of mutation rates compared with the higher-DMS subgroup, whereas the KRAS mutation rate increased in the high-DMS subgroup. TP53 and KRAS are prevalent oncogenic drivers in most tumor types, and their cooccurring mutations result in the upregulation of tumor immunogenicity and immune tolerance/escape in response to PD-1 blockade immunotherapy in LUAD (51). Furthermore, TTN and TP53 mutations may exhibit a synergistically prognostic benefit in various lung cancers except LUAD (52). Those findings supported that the notion that the DMS signature of DNA methylation regulator patterns, combined with gene mutation signals, as promising to predict the efficacy of anti-CTLA-4/PD-1/PD-L1 immune checkpoint blockade therapy, and could contribute to guide more effective strategies for precision immunotherapy in cancer individuals. Therefore, the DMS signature could stand as an important biomarker to estimate the benefit of antitumor immune response to cancer treatment (53, 54).

However, there were some limitations in this study. Firstly, we used only 21 DNA methylase-related regulators to construct the model system; newer methylation regulators should be included in further analyses. Second, additional clinical data are needed to validate the efficacy of the immunotherapy and to evaluate the accuracy of the DMP and DMS system.

## Conclusion

We identified two DNA methylation modification patterns in LUAD patients based on 21 methylase-related regulators and constructed a methylation profile score model for individual patients. The TME disparity between the distinct DMPs highlighted the potential complexity and heterogeneity of LUAD formation, casting light on multiple processes fueling tumor evolution, immune infiltration, and drug resistance. The systematic analysis of the immune and/or clinical characteristics in the DMP and DMS will contribute to enhance a deeper understanding of the tumor immune-infiltrating microenvironment and will promote the development of effectively targeted immunotherapies.

## Data Availability Statement

The original contributions presented in the study are included in the article/**Supplementary Material**. Further inquiries can be directed to the corresponding author.

## Author Contributions

DY designed the experiment, analyzed the data, drafted the manuscript, and coordinated the study. ZW revised the paper. YW, FC, LY, and SZ performed the table and figure processing. RT and JL supervised the study. All authors contributed to the article and approved the submitted version.

## Conflict of Interest

The authors declare that the research was conducted in the absence of any commercial or financial relationships that could be construed as a potential conflict of interest.

## Publisher’s Note

All claims expressed in this article are solely those of the authors and do not necessarily represent those of their affiliated organizations, or those of the publisher, the editors and the reviewers. Any product that may be evaluated in this article, or claim that may be made by its manufacturer, is not guaranteed or endorsed by the publisher.

## Supplementary Material

Supplementary Figure 1 Correlation and prognostic value of 21 DNA methylation regulators and overview of study design. **(A)** Overview of this study flow. **(B)** The cooccurrence and mutual exclusion of 21 regulators. Cooccurrence, blackish green; mutual exclusive, yellowish brown. **(C, D)** Clinical prognostic significance of expression of DNA regulators by univariate **(C)** and multivariate **(D)** Cox regression analyses. **(E–H)** Survival analysis of the regulators in TCGA cohort, including MBD3, DMAP1, and ZBTB38.

Supplementary Figure 2 Unsupervised clustering of 586 DNA methylation gene signatures and construction of the DMS system. **(A–C)** The consensus matrix (CM) plot of DNA methylation gene signature for *k* = 3 to *k* = 5. **(D)** The distribution of the consensus index cumulative distribution function (CDF) curves in the LUAD cohort for each *k*. **(E)** Tracking plot of subclusters at each *k*. **(F)** Correlation between DMS and immune-infiltrating trait in TCGA samples by the Spearman’s analysis. **(G)** Subcluster analysis determining the clinical prognostic ability of DMS in the meta-GEO cohort by multivariate Cox regression. The length of the horizontal line represented the 95% confidence interval for each group. **(H)** Survival analysis of patient between low- or high-DMS subclusters in the meta-GEO cohort. **(I)** The abundance of immune cell infiltration in different DMP groups using the ssGSVA algorithm in the meta-GEO cohort. **(J)** The methylation heatmap analysis of low- or high-DMS group.

Supplementary Table 1 Clinical information of LUAD cohorts from GEO/TCGA in this study.

Supplementary Table 2 Summary of DNA methylation modification regulators.

Supplementary Table 3 Clinical annotation and DNA methylation regulators of individual patient in TCGA-LUAD cohort.

Supplementary Table 4 Information of samples clustering in TCGA-LUAD cohorts.

Supplementary Table 5 Enrichment score of KEGG pathways in TCGA-LUAD cohorts.

Supplementary Table 6 Prognostic DEGs related to DNA methylation modification patterns using the univariate Cox regression model.

## Abbreviations

CNV, copy number variation; DEGs, differentially expressed genes; DMP, DNA methylation modification pattern; DMPs, DNA methylation patterns; DMS, DNA methylation score; GEO, Gene-Expression Omnibus; GO, Gene Ontology; GSVA, gene-set variation analysis; ICIs, immune checkpoint inhibitors; LUAD, lung adenocarcinoma; PCA, principal component analysis; ssGSEA, single sample gene-set enrichment analysis; TCGA, The Cancer Genome Atlas; TCIA, Cancer Immunome Database; TME, tumor microenvironment; TMB: tumor mutation burden; TPM, transcripts per kilobase million.
