# Supplementary figures and images for: DNA Methylation Regulator-Meditated Modification Patterns Define the Distinct Tumor Microenvironment in Lung Adenocarcinoma
Source: Front Oncol. 2021 Sep 6;11:734873. doi: 10.3389/fonc.2021.734873 (PMC8450540; doi:10.3389/fonc.2021.734873)

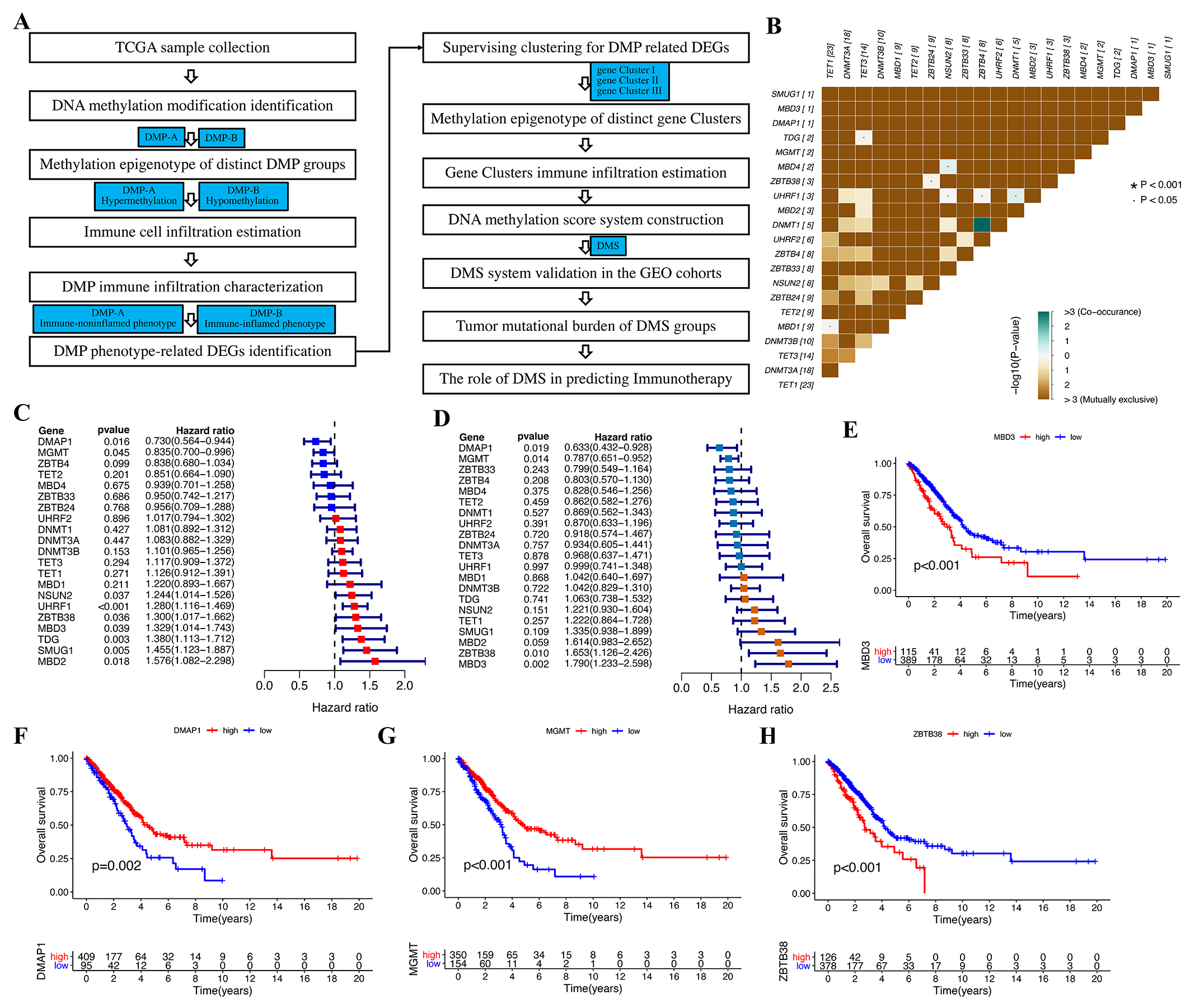

Supplement: Supplementary Figure 1 — Correlation and prognostic value of 21 DNA methylation regulators and overview of study design. (A) Overview of this study flow. (B) The cooccurrence and mutual exclusion of 21 regulators. Cooccurrence, blackish green; mutual exclusive, yellowish brown. (C, D) Clinical prognostic significance of expression of DNA regulators by univariate (C) and multivariate (D) Cox regression analyses. (E–H) Survival analysis of the regulators in TCGA cohort, including MBD3, DMAP1, and ZBTB38. [file Image_1.tif]

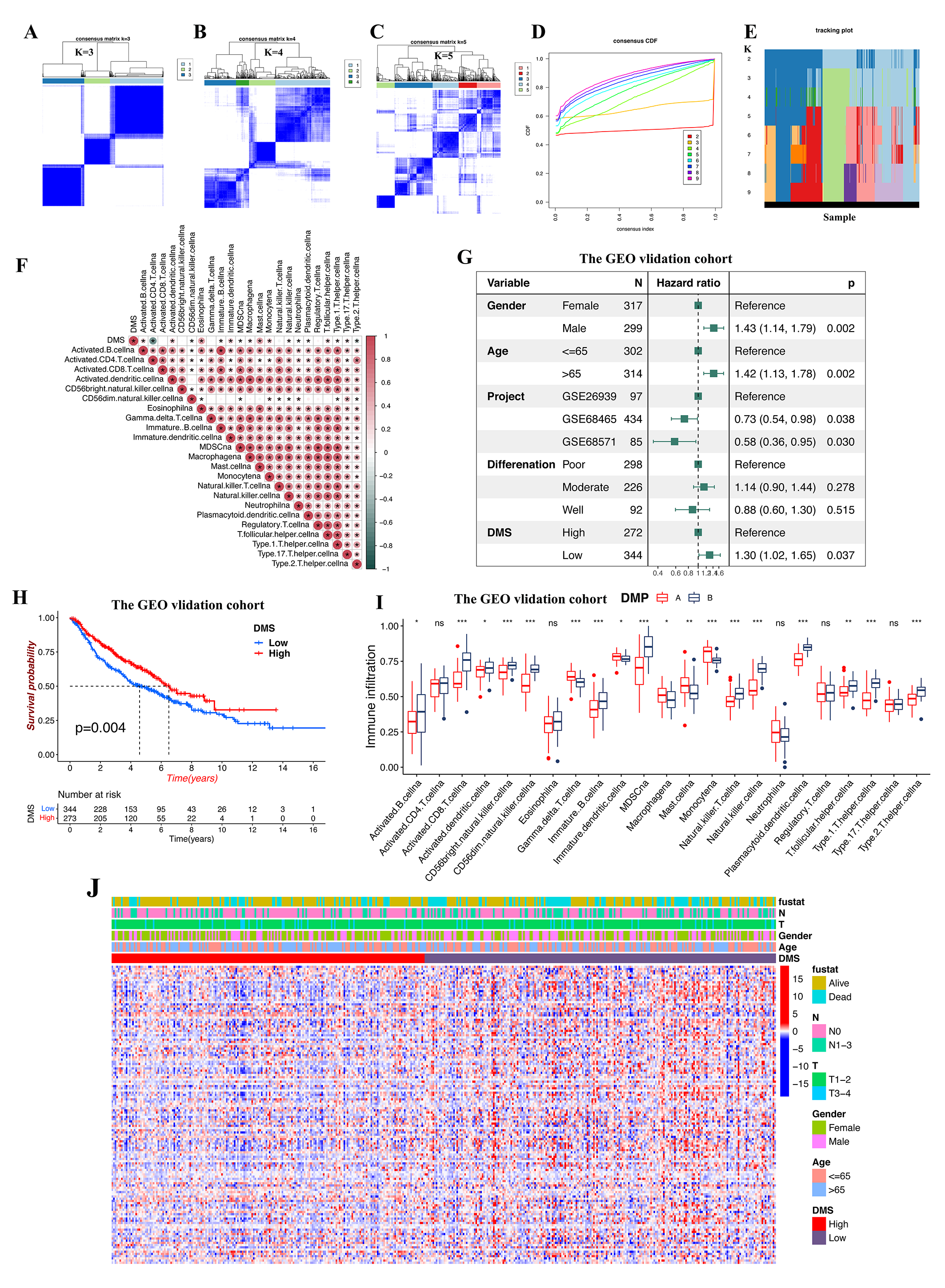

Supplement: Supplementary Figure 2 — Unsupervised clustering of 586 DNA methylation gene signatures and construction of the DMS system. (A–C) The consensus matrix (CM) plot of DNA methylation gene signature for k = 3 to k = 5. (D) The distribution of the consensus index cumulative distribution function (CDF) curves in the LUAD cohort for each k. (E) Tracking plot of subclusters at each k. (F) Correlation between DMS and immune-infiltrating trait in TCGA samples by the Spearman’s analysis. (G) Subcluster analysis determining the clinical prognostic ability of DMS in the meta-GEO cohort by multivariate Cox regression. The length of the horizontal line represented the 95% confidence interval for each group. (H) Survival analysis of patient between low- or high-DMS subclusters in the meta-GEO cohort. (I) The abundance of immune cell infiltration in different DMP groups using the ssGSVA algorithm in the meta-GEO cohort. (J) The methylation heatmap analysis of low- or high-DMS group. [file Image_2.tif]
